# A Large Ameloblastic Fibro-odontoma of the Maxillary Sinus

**Published:** 2014-04

**Authors:** Seyed Ali Banihashem Rad, Hamed Mortazavi, Majid Eshghpour, Jahanshah Salehinejad, Reza Shahakbari

**Affiliations:** 1 *Mashhad Dental Research Center, Department of Periodontology, Mashhad University of Medical Sciences Mashhad, Iran.*; 2 *Department of Oral Medicine, Shahid Beheshti Dental School, Tehran University of Medical Sciences Tehran, Iran.*; 3 *Mashhad Dental Research Center, Department of Oral and Maxillofacial surgery, Mashhad University of Medical Sciences, Mashhad, Iran.*; 4 *Mashhad Dental Research Center, Department of Oral and Maxillofacial Pathology, Mashhad University of Medical Sciences, Mashhad, Iran.*

**Keywords:** Ameloblastic fibro-odontoma, Maxillary sinus, Odontogenic tumor

## Abstract

**Introduction:**

Ameloblastic fibro-odontoma is a rare, benign, asymptomatic tumor. The term ameloblastic fibro-odontoma was first used by Hooker in 1967 as a separate lesion from ameloblastic odontoma.

**Case Report:**

This case report describes an eleven years old female with large ameloblastic fibro-odontoma in the right maxillary sinus.

**Conclusion:**

There is a low potential for recurrence after complete Enucleation of ameloblastic fibro-odontoma, but due to the risk of ameloblastic sarcoma after recurrence, the surgery should be perfect along with a careful follow up.

## Introduction

Ameloblastic fibro-odontoma (AFO) is a very rare, benign, asymptomatic, slow growing, expansile mixed odontogenic tumor (1). Odontogenic tumors constitute 0.84% to 1.78% of the histopathological results of oral pathology departments (2). Furthermore, odontogenic tumors account 7% of all oral lesions found in children and adolescents (3). AFO represents 1%-3% of all odontogenic tumors, reaching 4.6% when only the cases in children are mentioned, and 7.9% of odonto-genic tumors expect odontoma (1). The term ameloblastic fibro-odontoma was first used by Hooker in 1967 as a separate lesion from ameloblastic odontoma (4). Reichart and Ries classified this tumor as an ameloblastic ectomesenchymal tumor in 1983 (5). AFO has been also defined by who as "a neoplasm composed of proliferating odontogenic epithelium embedded in a cellular ectomesen- chymal tissue that resembles dental papilla with varying degree at inductive change and dental hard tissue formation" (6). It usually occurs in persons less than 20 years old with mean age11.5 years. Most cases are diagnosed between 9 and 11 years old. There is a higher incidence of AFO in men than women. The male to female ratio was reported 1.4:1,1.6:1 and 1.7:1 by Sassi, Boxberger and Minderjahn, respectively (7). However, Hutt did not show sex predilection (8). In a majority of cases, AFO arises in the posterior mandible and is usually associated with an unerupted tooth (1). Involvement of maxillary bone was also reported by Zouhary, Nouri and Miller (9). According to the review of the literature, AFO of the maxillary sinus is an extremely rare which was described by few authors such as Dolanmaz, Nouri and Ozer (10-11). 

Radiographically, AFO presents as a well-defined radiolucency containing radiopaque areas (11). The aim of this article is to report a case of massive AFO in the maxillary sinus. Due to the risk of recurrence, as well as becoming a fibrosarcoma, in the absence of proper treatment or inadequate follow up, introducing the lesion (due to its rarity) seems to be necessary (12).

## Case Report

An 11-year-old girl was referred to the Department of Oral and Maxillofacial Surgery, Mashhad Dental School, Iran for evaluation of a facial swelling of 6 months duration. There was no history of systemic disease and trauma. The extra oral examin- ation revealed an asymptomatic swelling on the right side of the maxilla without signs and symptoms of inflammation.

**Fig 1:**
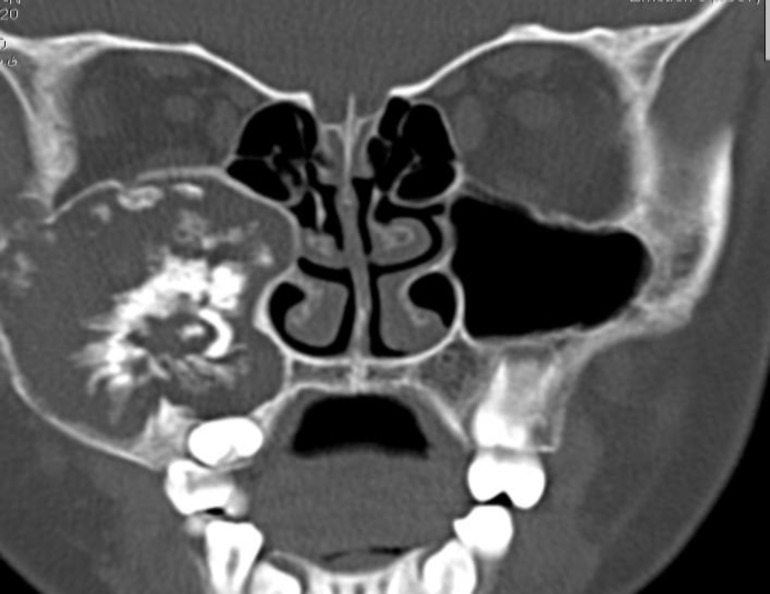
The coronal CT scan demonstrates a very large mixed tumor in the right maxillary sinus. Notice to the size of the lesion and extending to eye floor and wall of the nose

Intra-orally, a bony hard bulge was palpable in the maxillary vestibule. A full complement of the teeth with the exception of missing second molar was marked able. Oral mucosa was normal and tooth mobility was not seen. Radiographically, computerized tomography scan (CT) showed a well-defined, radiolucent lesion in the maxillary sinus which contained several radiopaque materials of varying sizes and shapes (Fig. 1).

**Fig 2:**
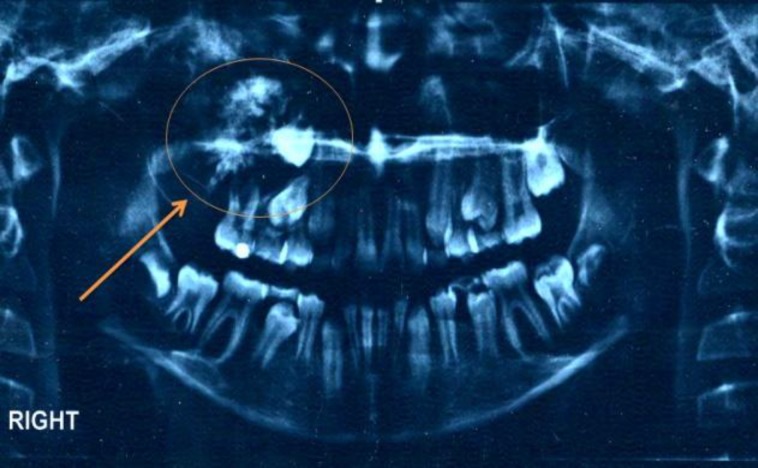
The panoramic view of the patient before surgery shows a very large mixed tumor in the right maxillary sinus. The maxillary second molar is involved in the lesion

The panoramic view showed a very large lesion which the maxillary second molar had been involved (Fig.2). 

As the clinical and radiological presentations alone could not show a definitive diagnosis, incisional biopsy was performed. Specimen was sent to the department of oral and maxillofacial pathology. Pathologist reported: this lesion is lobulated in general configuration and is surrounded by a fibrous capsule.

**Fig 3:**
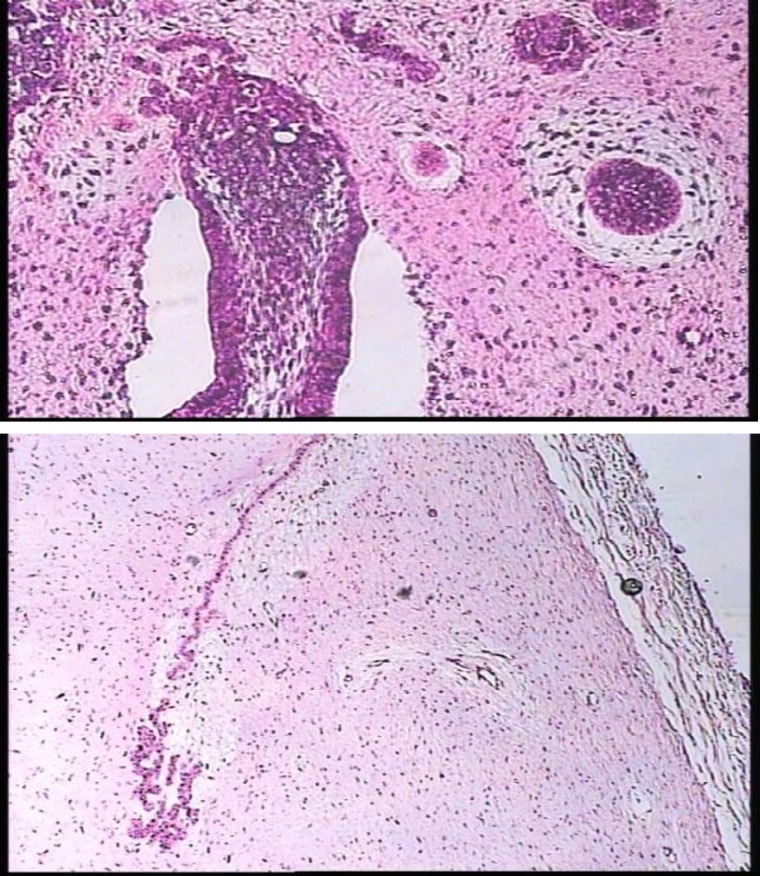
Microscopic view of the lesion shows enamel and dentin similar to compound and complex odontoma and epithelial component. The tumor mass is composed predominantly of a primitive appearing myxoid connective tissue similar to dental pulp

The tumor mass is composed predominantly of a primitive appearing myxoid connective tissue similar to dental pulp. The epithelial component has been compared microsco- pically to the dental lamina that proliferates from oral epithelium in the early stages of the tooth developments. Some foci contain enamel and dentin similar to compound and complex odontoma (Fig. 3).

These findings were consistent with diagnosis of ameloblastic fibro-odontoma.

**Fig 4:**
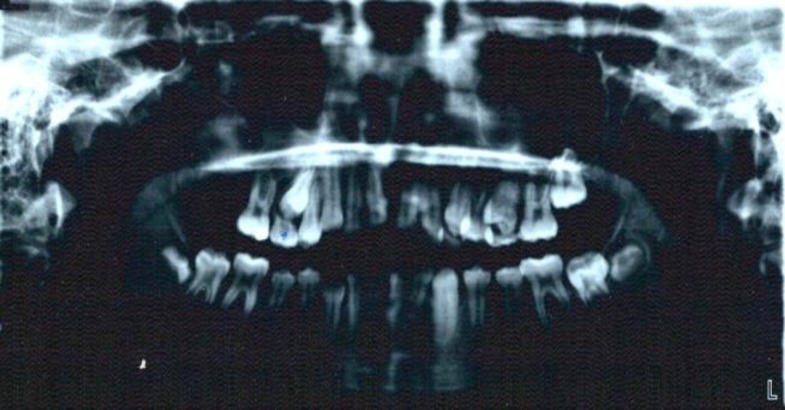
The panoramic view of the patient 4 weeks after surgery

**Fig 5:**
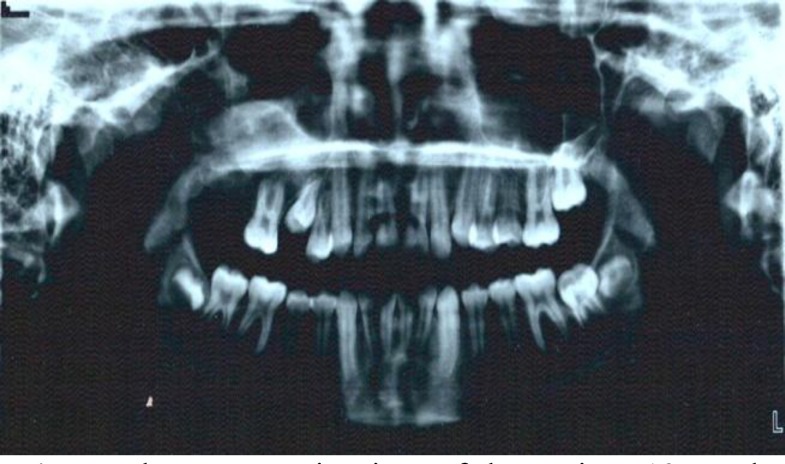
The panoramic view of the patient 12 weeks after surgery.The right second premolar is in eruption

After a final histological diagnosis, the patient was submitted for surgical excision and enucleation of the lesion. The patient was taken to the operation room, under general anesthesia an incision was made intraorally. A full thickness mucoperiosteal flap from second incisor to the tuberosity was reflected. After bone removal of the sinus wall, access to the lesion was completed. The lesion and the impacted second molar were enucleated Finally, histopathological examination of the last specimen confirmed the diagnosis of AFO. Postoperatively, after twelve months, no evidence of residual or recurrent disease was found (Fig.4,5).

## Discussion

There are different concepts about the nature of AFO in the literature. Regezi demonstrated that AFO is a derivative of the ameloblastic fibroma (12). Slootweg described that the AFO is an immature complex odontoma (13). Neoplastic behavior and malignant features of AFO has been also reported by Howell and Bregni (14). The most common clinical presentations of AFO are asymptomatic swelling and failure of tooth eruption (12). 

The pathologically and radiologically differential diagnosis of AFO are included: ameloblastoma, odontogenic myxoma, dentinogerous cyst, odontogenic keratocyst, central giant cell granuloma, histocytosis-X group of lesions, calcifying odontogenic cyst, calcifying epithelial odontogenic tumor, adenomatoid odontogenic tumor and immature odontoma (12).

There is controversy over the management of AFO. According to the literature, conservative surgical excision is an accepted treatment for this lesion. In most cases, the impacted tooth associated with the tumor is removed at the same time (14). 

There is a low potential for recurrence. According to Boxberger, in almost all cases recurrences were related to incomplete removal of the lesion at the time of the initial surgery.

## Conclusion

Ameloblastic fibro-odontoma (AFO) is a very rare, benign, asymptomatic, slow growing, expansile mixed odontogenic tumor. Note that this tumor is encapsulated, treatment plan is surgical excision. Despite the little potential of this lesion to locally recur, due to a low risk for ameloblastic sarcoma, a careful follow up is recommended.
